# Adiponectin Increases Secretion of Rat Submandibular Gland via Adiponectin Receptors-Mediated AMPK Signaling

**DOI:** 10.1371/journal.pone.0063878

**Published:** 2013-05-07

**Authors:** Chong Ding, Li Li, Yun-Chao Su, Ruo-Lan Xiang, Xin Cong, Hong-Kui Yu, Sheng-Lin Li, Li-Ling Wu, Guang-Yan Yu

**Affiliations:** 1 Center for Salivary Gland Diseases and Center Laboratory, Peking University School and Hospital of Stomatology, Beijing, China; 2 Department of Physiology and Pathophysiology, Peking University Health Science Center and Key Laboratory of Molecular Cardiovascular Sciences, Ministry of Education, Beijing, China; 3 Department of Oral and Maxillofacial Surgery, Peking University School and Hospital of Stomatology, Beijing, China; Cincinnati Children's Hospital Medical Center, United States of America

## Abstract

Adiponectin and adiponectin receptors (AdipoR1/2) are expressed in various tissues and are involved in the regulation of multiple functions such as energy metabolism and inflammatory responses. However, the effect of adiponectin and AdipoRs in submandibular glands has not been fully evaluated. In the present study, we found that mRNA and protein of both adiponectin and AdipoR1/2 were expressed in rat submandibular glands and in the SMG-C6 cell line, as evidenced by RT-PCR and Western blot analysis. Immunofluorescence staining showed that adiponectin was diffused in the cytoplasm, while AdipoR1/2 was concentrated in the membrane of acinar cells. Saliva flow was significantly increased by full length adiponectin (fAd) or globular adiponectin (gAd) perfusion in isolated rat submandibular glands. 5-Aminoimidazole-4-carboxamide-1-4-ribofuranoside (AICAR), an adenosine monophosphate activated protein kinase (AMPK) activator, also increased saliva secretion. fAd, gAd, and AICAR all increased the average width of apical tight junctions in perfused submandibular glands, and decreased transepithelial electrical resistance (TER) in SMG-C6 cells, suggesting that adiponectin promoted secretion by modulating paracellular permeability. fAd and gAd increased p-AMPK levels, while AraA, an AMPK antagonist, abolished fAd- and gAd-induced changes in secretion, tight junction ultrastructure, and TER. Moreover, both AdipoR1 and AdipoR2 were required for fAd- or gAd-induced p-AMPK and TER responses, suggesting from their inhibition following AdipoR1 or AdipoR2 knockdown, and co-knockdown of AdipoRs by RNA interference. Our results suggest that adiponectin functions as a promoter of salivary secretion in rat submandibular glands via activation of AdipoRs, AMPK, and paracellular permeability.

## Introduction

Adiponectin is an adipokine mainly derived from white adipose tissue and exists abundantly in circulating blood [1]. Human adiponectin is defined as a 244-amino acid polypeptide with a collagen-like domain, and a C1q-like globular domain. The full-length adiponectin (fAd) is the primary circulating form, and its proteolytically cleaved globular domain at the carboxyl terminal, known as globular adiponectin (gAd), is the biologically active domain [2]. Many studies have implicated that adiponectin is involved in the regulation of energy metabolism and inflammatory responses [3]. In addition, adiponectin is also considered an important endogenous protective adipokine against obesity, diabetes, atherosclerosis, and cardiovascular diseases [4]–[6]. Circulating adiponectin levels are lower in patients with obesity, type 2 diabetes, and coronary artery disease. Hypoadiponectinemia may be a biomarker for metabolic and cardiovascular diseases [7]–[9].

Two transmembrane proteins have been identified as receptors for adiponectin (AdipoRs), AdipoR1 and AdipoR2, which mediate the primary function of adiponectin via the activation of adenosine monophosphate activated protein kinase (AMPK), as well as the subsequent fatty acid oxidation and glucose uptake [10]. Expressed ubiquitously among tissues, AdipoR1 is most concentrated in skeletal muscle, and functions as a high-affinity receptor for gAd and a low-affinity one for fAd. AdipoR2 is expressed primarily in the liver and acts as an intermediate-affinity receptor for both gAd and fAd [11]. In addition, T-cadherin has been identified as the third adiponectin receptor, which is exclusively expressed in endothelial and smooth muscle cells [12]. As a critical component in the adiponectin signaling cascade, downregulation of AdipoRs may have an important impact on the development and progression of metabolic and cardiovascular diseases [13], [14].

Saliva secretion is mainly controlled by parasympathetic and sympathetic autonomic nerves and the related receptors [15]. In addition, some peptides, such as substance P and neuropeptide Y, are also involved in regulating saliva secretion through their respective receptors [16]. Recent research has shown that the expression of adiponectin and AdipoRs are detectable in salivary glands [17]–[19]. In addition, the level of adiponectin secreted from minor salivary gland epithelial cells is increased in patients with primary Sjögren's syndrome, and it inhibits cell apoptosis induced by interferon-γ [19], [20]. These results suggest that adiponectin and its receptors expressed in salivary glands may be involved in the regulation of local inflammatory and immunological processes. However, whether adiponectin is involved in the regulation of saliva secretion is unknown.

Therefore, the present study was designed to explore the following: (1) the expression and distribution of adiponectin and AdipoRs in rat submandibular glands; (2) the effect of adiponectin on secretory function of submandibular glands; and (3) the possible molecular mechanism involved in the regulation of saliva secretion by adiponectin.

## Materials and Methods

### Materials

Human recombinant fAd and gAd were purchased from Phoenix Pharmaceuticals Inc. (San Francisco, CA, USA). 5-Aminoimidazole-4-carboxamide-1-4- ribofuranoside (AICAR), adenine 9-β-D-arabinofuranoside (AraA), and atropine were from Sigma-Aldrich (St. Louis, MO, USA). Antibodies to adiponectin, AdipoR1, AdipoR2, occludin, actin, and aquaporin 5 (AQP5) were from Santa Cruz Biotechnology (Santa Cruz, CA, USA). Antibodies to p-AMPK and AMPK were purchased from Cell Signaling Technology (Danvers, MA, USA). Antibody to glyceraldehyde-3-phosphate dehydrogenase (GAPDH) was from Abmart (Shanghai, China). IgG horseradish peroxidase (HRP)-conjugated, and FITC-, TRITC-conjugated secondary antibodies were from ZSGB-BIO (Beijing, China).

### Perfusion of rat isolated submandibular glands

Healthy male Sprague Dawley (SD) rats, weighing 250–270 g each, were obtained from Peking University Health Science Center. All experimental procedures were approved by the Peking University Institutional Review Board, for the care and use of laboratory animals. All surgical procedures were performed under chloral hydrate anesthesia, and all efforts were made to minimize suffering.

The effect of adiponectin on the secretory function in submandibular glands was measured according to the methods previously described [21]. Briefly, under chloral hydrate (400 mg/kg body weight) anesthesia, submandibular glands were isolated and perfused through a polyethylene cannula placed in the external carotid artery. The main excretory duct was cannulated for saliva collection. Krebs-Ringer-HEPES (KRH, 116 mM NaCl, 5.4 mM KCl, 1.25 mM CaCl_2_, 0.4 mM MgSO_4_, 20 mM HEPES, 0.9 mM Na_2_HPO_4_, and 5.6 mM glucose, pH 7.4) buffer was warmed to 37°C, bubbled with 95% O_2_ and 5% CO_2_, and perfused to the glands at a rate of 1.8 ml/min with a Gilson Minipuls rotary pump. After equilibration for at least 30 min, different stimulators or inhibitors (n = 8 for each group) were perfused into the glands for 10 min, respectively. The secretion of the glands was measured as the length of moisture on the filter paper (35 mm×5 mm) after perfusion of drugs. In each group, perfused glands were collected after drug perfusion and analyzed by Western blot method and transmission electron microscopy. The other 4 glands were then washed with KRH for 10 min, and their salivary flow rates were measured again.

### Cell culture

The rat submandibular gland cell line SMG-C6 (a generous gift from Dr. David O. Quissell without commercial purpose; ref: 22) was routinely grown at 37°C in a humidified 5% CO_2_ atmosphere in DMEM/F12 (1∶1 mixture) medium containing 2.5% fetal bovine serum, 5 μg/ml transferrin, 1.1 μM hydrocortisone, 0.1 μM retinoic acid, 2 nM thyronine T3, 5 μg/ml insulin, 80 ng/ml epidermal growth factor, 50 μg/ml gentamicin sulfate, 5 mM glutamine, 100 U/ml penicillin, and 100 μg/ml streptomycin [22]. All constituents used in culturing SMG-C6 cells were purchased from Sigma-Aldrich Co.

### Total RNA extraction and RT-PCR

Total RNA was purified with Trizol (Invitrogen, Carlsbad, CA, USA) according to the manufacturer's instructions. First-strand cDNA was reversed according to the RevertAid First Strand cDNA Synthesis Kit (Promega, Madison, WI, USA) with 2 μg of total RNA. PCR was performed with PCR Master Mix (Promega, Madison, WI, USA), and the primer sequences were as follows: adiponectin (NM_144744), 5′-GAATCATTATGACGGCAGCAC-3′ and 5′-CTTGGAGCCAGACTTGGTCTC-3′; AdipoR1 (NM_207587), 5′-AGGCAACTGCTGGTCCTTCAC-3′ and 5′-TGCCAAGCGGTCTGTACTTTC-3′; AdipoR2 (NM_001037979), 5′-AACCCACAACCTTGCTTCATC-3′ and 5′-TCACAGCGCATCCTCTTCAGT-3′; GAPDH (NM_017008), 5′-ACAGCAACAGGGTGGTGGAC-3′ and 5′-TTTGAGGGTGCAGCGAACTT-3′. The products were separated by electrophoresis on a 1.5% agarose gel, and the DNA bands were visualized by staining with ethidium bromide.

### Western blot analysis

The submandibular gland tissues and cultured cells were homogenized with lysis buffer (containing 50 mM Tris-HCl, 150 mM NaCl, 1 mM EDTA, 1 mM phenylmethylsulfonyl fluoride, 1% Triton X-100, 0.1% SDS, and 0.1% sodium deoxycholate, pH 7.2) using a polytron homogenizer as previously described [23]. The homogenates were centrifuged at 1000 g for 10 min at 4°C. The membrane and cytosol fractions of SMG-C6 cells were extracted with Membrane and Cytosol Protein Extraction Kit according to the manufacturer's instructions (Beyotime Institute of Biotechnology, Nantong, China). Protein concentration of the supernatant was measured by the Bradford method. Equal amounts of proteins (20 μg) derived from each sample were separated on 9% SDS-PAGE and electroblotted on polyvinylidene fluoride membranes. Non-specific binding was blocked with 5% non-fat milk in PBS-T (137 mM NaCl, 2.7 mM KCl, 4.3 mM Na_2_HPO_4_, 1.4 mM KH_2_PO_4_, 0.1% Tween-20, pH 7.4) for 1 h at room temperature. Blocked membranes were incubated overnight at 4°C with antibodies against adiponectin (1∶500), AdipoR1 (1∶800), AdipoR2 (1∶800), p-MAPK (1∶1000), AMPK (1∶1000), AQP5 (1∶500), or GAPDH (1∶4000), respectively. The membranes were respectively then probed with HRP-conjugated secondary antibodies (1∶4,000–8,000) at room temperature for 1 h. Immunoreactive protein bands were visualized by use of an enhanced chemiluminescence detection system (GE Biosciences, Buckinghamshire, England) according to the manufacturer's protocol. Densitometry data were analyzed by Quantity One (Bio-Rad, Hercules, CA, USA).

### Immunofluorescence

Gland tissues were sectioned 5 μm thick and fixed in cold acetone for 15 min as previously reported [23]. SMG-C6 cells were plated on coverslips and fixed in 4% paraformaldehyde for 10 min. After washing 3 times with PBS, tissue slides and cells were blocked with 20% rabbit serum for 30 min, immunostained with the antibodies against adiponectin (1∶100), AdipoR1 (1∶100), AdipoR2 (1∶100), occludin (1∶100), calponin (1∶100), and AQP5 (1∶100) overnight at 4°C, and then incubated with FITC- and TRITC-conjugated secondary antibodies (1∶200) for 1 h. Nuclei were labeled with 4, 6-diamidino-2-phenylindole (DAPI). Fluorescence images were captured on a confocal microscope (Leica TCS SP5, Wetzlar, Germany).

### Transmission electron microscopy

The gland specimens were fixed in 2% paraformaldehyde-1.25% glutaraldehyde. Ultrathin sections were stained with uranyl acetate and lead citrate, and examined with a transmission electron microscope (H-7000 electron microscope, HITACHI, Tokyo, Japan). Each image was taken under the same conditions, such as brightness and contrast, for a better comparison on tight junction density among the different groups. For morphometric analysis as previously described [24], the distance between neighboring tight junctions (shown as the width of apical tight junctions) from 4 sections of 10 randomly selected fields in each section were measured and averaged by the use of ImageJ software (NIH) blindly by two examiners.

### Transepithelial electrical resistance measurement

Confluent monolayer of SMG-C6 cells were grown in 24-well Transwell^TM^ chambers (polycarbonate membrane, filter pore size: 0.4 μm; filter area: 0.33 cm^2^; Costar, USA) for 5 to 7 days before they were used for experiments. Transepithelial electrical resistance (TER) was measured at 37°C using an EVOM (Epithelial Volt Ohm Meter; WPI, FL, USA) as described previously [25]. The value of the blank filter (90 Ω) was subtracted, and all measurements were performed with a minimum of triplicate wells.

### shRNA knockdown

SMG-C6 cells were cultured to 80% confluence and transfected with shRNA of interests by use of MegeTran 1.0 (Origene, MD, USA) according to the manufacturer's instructions. For knockdown of AdipoR1 and AdipoR2, shRNA constructs against rat AdipoR1 5′-CGTCTACTGTTCAGAGAA-3′, AdipoR2 5′-CCTTGCTTCATCTACCTGATT-3′, and scrambled control were constructed in pGFP-V-RS vectors and synthesized by Origene Technologies (Origene, MD, USA). After transfection for 48 h, the cells were collected for Western blot analysis.

### Statistical analysis

Data are presented as means ± SD. Statistical analysis among multiple groups was performed by one-way ANOVA and followed by Bonferroni's test using GraphPad software (GraphPad Prism, CA, USA). *P*<0.05 was considered significant.

## Results

### Adiponectin, AdipoR1, and AdipoR2 are expressed in rat submandibular glands and SMG-C6 cells

**Figure 1:**
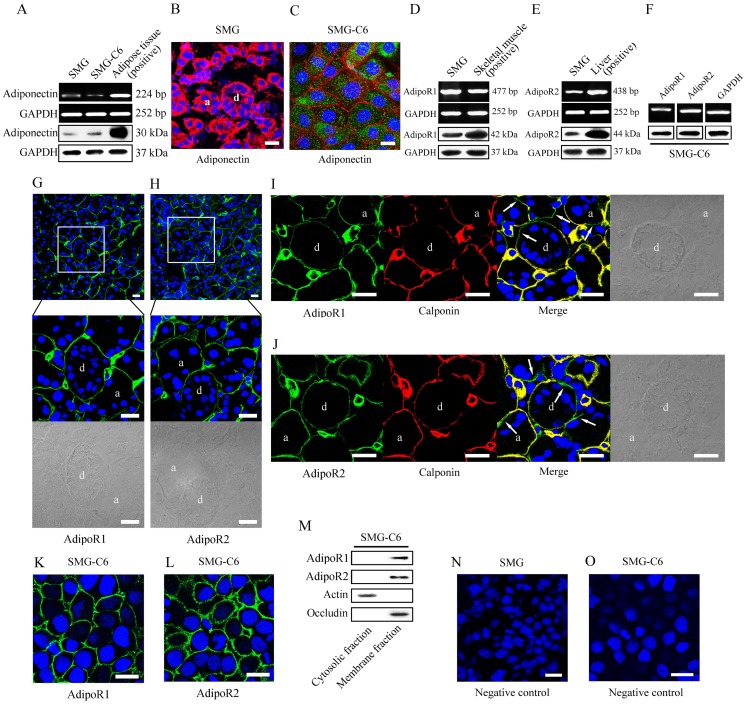
Expression and distribution of adiponectin and AdipoRs in rat submandibular glands and SMG-C6 cells. (A) Expression of adiponectin mRNA and protein in rat submandibular glands and SMG-C6 cells was detected by RT-PCR and Western blot analysis with epididymal adipose as positive control. GAPDH mRNA and protein expressions were used as internal controls. SMG: submandibular gland. (B) Representative immunofluorescence image of adiponectin in rat submandibular glands. Gland tissues were sectioned, fixed, and incubated with anti-adiponectin antibody, then stained with a TRITC-conjugated secondary antibody. Nuclei stained with DAPI (blue) were shown in all images in Figure 1. a, acinus; d, duct. Bar: 20 μm. (C) Representative immunofluorescence images of adiponectin (green) and occludin (red) in SMG-C6 cells. Bar: 20 μm. (D and E) Expression of AdipoR1 and AdipoR2 mRNA and protein in rat submandibular glands with skeletal muscle and liver as positive control of AdipoR1 and AdipoR2, respectively. GAPDH mRNA and protein expressions were used as internal controls. (F) Expression of AdipoR1 and AdipoR2 mRNA and protein in SMG-C6 cells. Representative immunofluorescence images AdipoR1 (G) and AdipoR2 (H) in submandibular glands with DIC images. a, acinus; d, duct. Bar: 20 μm. (I) Co-staining of AdipoR1 (green) and calponin (red) in submandibular glands. AdipoR1 positive staining without calponin expression was shown with arrows in the merged picture. Bar: 20 μm. (J) Co-staining of AdipoR2 (green) and calponin (red) in submandibular glands. AdipoR2 positive staining without calponin expression was shown with arrows on the merged picture. Bar: 20 μm. Distribution of AdipoR1 (K) and AdipoR2 (L) in SMG-C6 cells. Bar: 20 μm. Expression of AdipoR1 and AdipoR2 in cytosolic and membrane fractions of SMG-C6 cells (M). Expressions of actin and occludin were used as the internal controls for the cytosolic and the membrane fractions, respectively. The negative control in gland tissues (N) and SMG-C6 cells (O). Bar: 20 μm.

As shown in Fig. 1A, the expression of adiponectin mRNA was detected at the expected size of 224 bp and adiponectin protein was observed with a molecular mass of approximately 30 kDa in rat submandibular glands and SMG-C6 cells. Rat epididymal adipose served as the positive control. Immunofluorescence staining showed that adiponectin (red) was widely diffused in the cytoplasm of acinar and duct cells in gland tissues (Fig. 1B). In SMG-C6 cells, adiponectin (green) was dispersed in the cytoplasm (Fig. 1C). Occludin (red) was used as a membrane marker [26], which encircled the cells and delineated the cellular boundaries of SMG-C6.

Expressions of AdipoR1 and AdipoR2 mRNA were detected at the expected size of 477 bp and 438 bp, and their proteins were observed with a molecular mass of approximately 42 kDa and 44 kDa in rat submandibular glands, respectively. Rat skeletal muscle and liver were used as positive controls for AdipoR1 and AdipoR2, respectively (Figs. 1D and E). AdipoR1 and AdipoR2 mRNA and protein expression were also confirmed in SMG-C6 cells (Fig. 1F). AdipoR1 and AdipoR2 were mainly located at the basal membrane of acinar cells in rat submandibular glands. Positive stainings were also observed at the basal membrane of most of the ductal cells (Figs. 1G and H). To identify whether the location of AdipoRs was related to myoepithelial cells, we further examined the distribution of calponin, a specific marker of myoepithelial cells [27]. Calponin expression (red) was mostly observed in the periphery of acini and ducts, which was consistent with the location of calponin in submandibular glands in previous report [27]. Although the merged images revealed that AdipoR1 or AdipoR2 were co-localized with calponin (yellow) in myoepithelial cells, the positive staining of AdipoR1 and AdipoR2 was also detected in acini and duct with negative staining of calponin (Figs. 1I and J). These results confirmed that AdipoR1 and AdipoR2 were expressed in acinar and duct cells as well as myoepithelial cells of submandibular glands. In SMG-C6 cells, immunofluorescence stainings of AdipoR1 and AdipoR2 were located in the cell membrane (Figs. 1K and L), and Western blot analysis results also confirmed that AdipoRs were expressed in membrane fraction, but not in cytosolic fraction of SMG-C6 cells (Fig. 1M). The expressions of actin and occludin were used as internal controls for the cytosolic and the membrane fractions, respectively. The negative control of gland tissues and SMG-C6 cells were shown in Figs. 1N and O. These results confirmed that adiponectin, AdipoR1, and AdipoR2 existed in rat submandibular glands and SMG-C6 cells.

### fAd and gAd promote secretion of rat isolated submandibular glands

**Figure 2:**
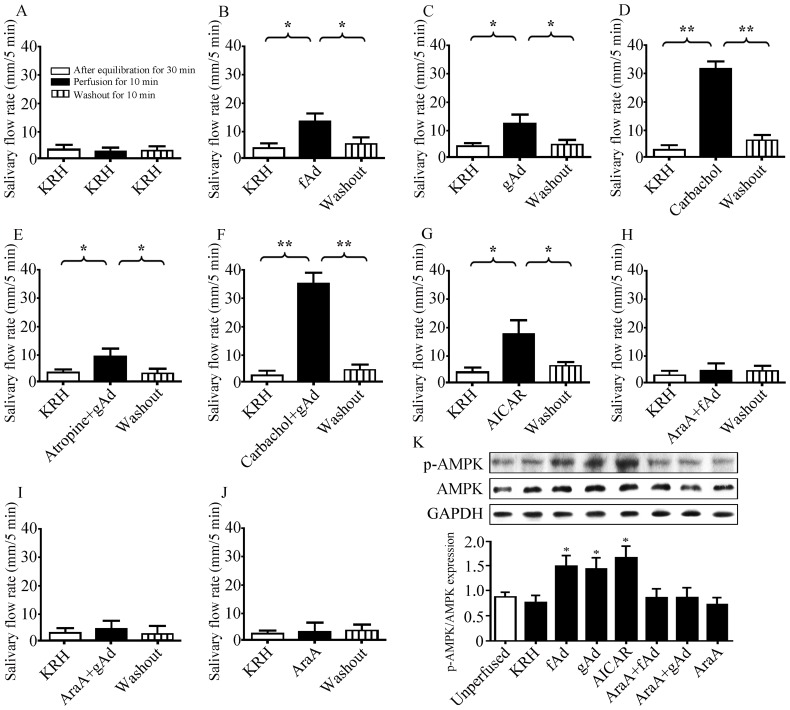
fAd and gAd promote secretion of rat isolated submandibular glands in an AMPK dependent manner. The isolated rat submandibular glands were perfused with KRH and salivary flow rates were measured after equilibration for 30 min, different stimulators or inhibitors perfusion for 10 min, and KRH washout for 10 min. (A) KRH perfusion for 50 min. (B) 5 μg/ml fAd. (C) 1 μg/ml gAd. (D) 1 μM carbachol. (E) Pre-perfused with 0.5 μM atropine for 30 min, then gAd for 10 min. (F) Carbachol combined gAd perfusion for 10 min. (G) 1 mM AICAR. Pre-perfusion with 1 mM AraA for 30 min, then perfused with fAd (H), gAd (I) or AraA alone (J). Values are means ± SD from 4 independent experiments. *P<0.05 and **P<0.01 compared with KRH perfusion. (K) p-AMPK expression in the different groups. GAPDH expression was used as an internal control. Values are means ± SD from 4 independent experiments. *P<0.05 compared with KRH group.

To identify the role of adiponectin in the secretion of submandibular glands, we performed *ex vivo* perfusion in isolated rat submandibular glands. The basal saliva flow was not changed within KRH perfusion for 50 min (Fig. 2A). Saliva flows were significantly increased by 294.95% with fAd (5 μg/ml) or 243.11% with gAd (1 μg/ml) after perfusion for 10 min, and returned to the basal level after washout with KRH for 10 min (Figs. 2B and C). Carbachol (1 μM), a muscarinic agonist, increased saliva flow rate by 1410.38%, which was used as the positive control (Fig. 2D). Pre-perfusion with atropine (0.5 μM), a muscarinic antagonist, for 30 min did not abolish the adiponectin-induced increase in saliva secretion (Fig. 2E). In addition, the flow rate was further increased by 1704.74% after perfusion by carbachol combined with gAd (Fig. 2F).

### Activation of AMPK is required by fAd- and gAd-induced secretion of submandibular glands

AMPK is one of the central mediators of adiponectin-derived biological effects. To further investigate the possible effect of AMPK on adiponectin-induced saliva secretion, AICAR (1 mM), a pharmacological agonist of AMPK, was perfused into the isolated glands. As shown in Figure 2G, AICAR increased the secretion by 351.11% (*P*<0.05). Pre-perfusion with 1 mM AraA, an AMPK antagonist, abolished the increased secretion induced by either fAd or gAd (Figs. 2H and I), whereas AraA alone had no influence on the basal salivary flow (Fig. 2J). We next detected AMPK phosphorylation in glands with drug perfusion for 10 min but without washing with KRH (Fig. 2K). The levels of p-AMPK in fAd, gAd, or AICAR-perfused glands were increased by 93.59% (*P*<0.05), 84.62% (*P*<0.05), and 116.67% (*P*<0.05), respectively, as compared with that in KRH perfused glands. Pre-perfusion with AraA abolished fAd- or gAd-induced AMPK phosphorylation, whereas AraA alone did not change the level of p-AMPK. These results suggested that adiponectin-elicited salivation in perfused submandibular glands was dependent on the activation of AMPK.

### Adiponectin did not change the expression and distribution of AQP5 in rat submandibular glands

**Figure 3:**
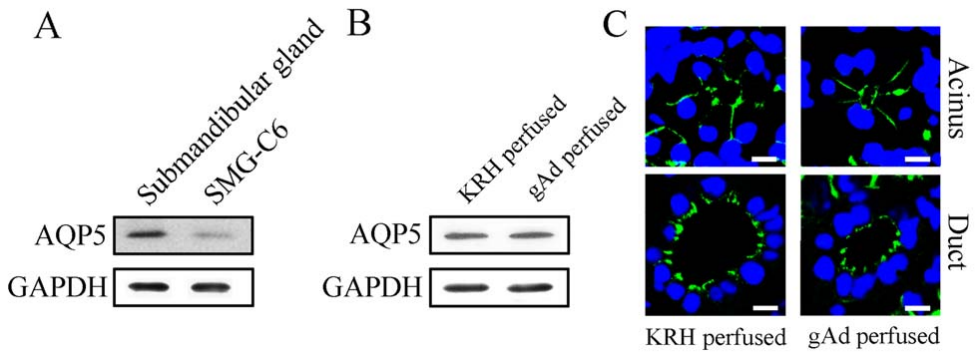
The effect of gAd on the expression and distribution of AQP5 in rat submandibular glands. The protein expression of AQP5 in rat submandibular glands and SMG-C6 cells (A). The protein expression of AQP5 in the perfused glands without and with gAd (B). The distribution of AQP5 in acinus and duct of the perfused glands without and with gAd. Bar: 10 μm.

AQP5 plays an important role in the rapid and transcellular movement of water in salivary glands [28]. To explore whether AQP5 was involved in adiponectin-induced saliva secretion, we measured the expression and distribution of AQP5. As shown in Fig. 3A, AQP5 protein was expressed in rat submandibular glands and SMG-C6 cells. In the perfused glands without gAd, AQP5 was mainly located in apical and lateral membrane of acinar cells, as well as the luminal side of the duct cells (Fig. 3C), consistent with previous studies [29], [30]. gAd (1 μg/ml) perfusion for 10 min did not change the expression and distribution of AQP5 in the perfused submandibular glands (Figs. 3B and C).

### fAd and gAd alter the ultrastructure of tight junction in the perfused submandibular glands

**Figure 4:**
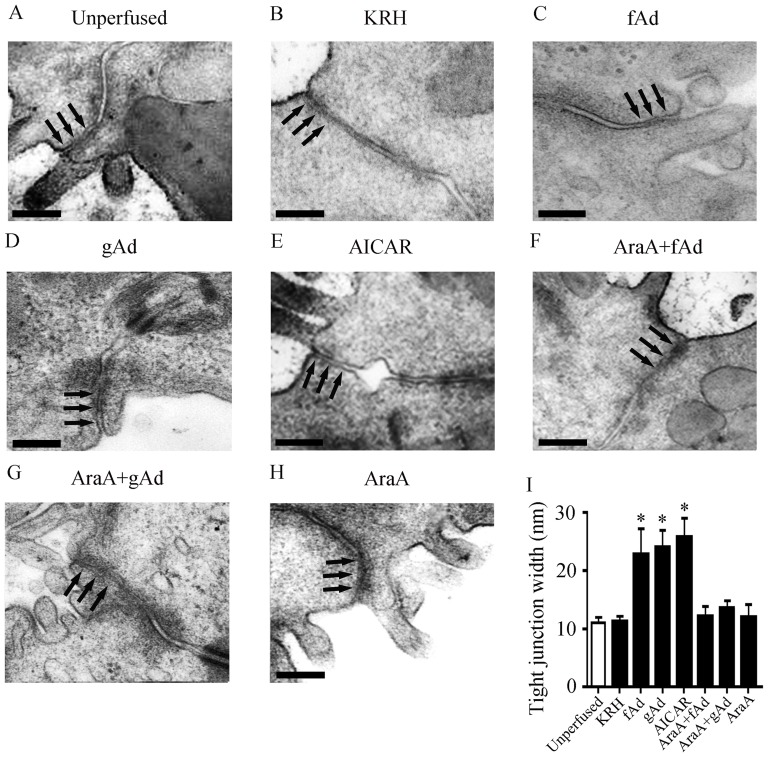
fAd and gAd alter the ultrastructure of tight junctions in the perfused submandibular glands. Representative images of tight junctions in submandibular glands without (A), or with KRH (B), 5 μg/ml fAd (C), 1 μg/ml gAd (D), or 1 mM AICAR (E) perfusion. The glands were pre-perfused with 1 mM AraA, then followed by fAd (F), gAd (G), or AraA alone (H). Tight junctions were showed with arrows. Bar: 200 μm. (I) The width of apical tight junctions was measured from 4 sections of 10 random fields in the above 8 groups. Values are means ± SD from 4 independent experiments. *P<0.05 compared with KRH group.

Tight junctions are cell-cell interactions ubiquitously expressed in epithelium and endothelium, playing an essential role in regulating water and solute transport through the paracellular pathway [31]. Since AMPK regulates the assembly of tight junctions in MDCK and Caco-2 cells [32]–[34], we next examined the effect of adiponectin on morphology of tight junctions. In submandibular glands without perfusion, tight junctions were located in the apical portion between neighboring epithelial cells, and formed a slightly dilated distance under transmitted electron microscope (Fig. 4A). The width of apical tight junctions is used as an indicator of tight junction structure [24], [35], [36]. The ultrastructure of tight junctions was not affected by KRH perfusion (Fig. 4B). However, the distance between the apical portion of neighboring epithelial cells was enlarged in the glands perfused with fAd (Fig. 4C), gAd (Fig. 4D), or AICAR (Fig. 4E). Pre-treatment with AraA suppressed the increased distance between apical tight junctions induced by fAd or gAd (Figs. 4F and G), while AraA alone had no influence on the ultrastructure of tight junctions (Fig. 4H). Quantitative analysis showed the average width of apical tight junctions was 12.28±0.05 nm in KRH-perfusion glands, which was similar with that in unperfused glands. fAd, gAd, and AICAR significantly increased the width of tight junctions by 89.09% (*P*<0.05), 102.04% (*P*<0.05), and 113.76% (*P*<0.05), respectively, as compared with that in the KRH group. Pre-perfusion with AraA suppressed the increased width of apical tight junctions induced by fAd and gAd, whereas AraA alone had no effect on the width of apical tight junctions (Fig. 4I). These results suggested that fAd and gAd might regulate the “open” of tight junction structure in an AMPK-dependent manner.

### fAd and gAd decrease TER value in an AMPK-dependent manner in SMG-C6 cells

**Figure 5:**
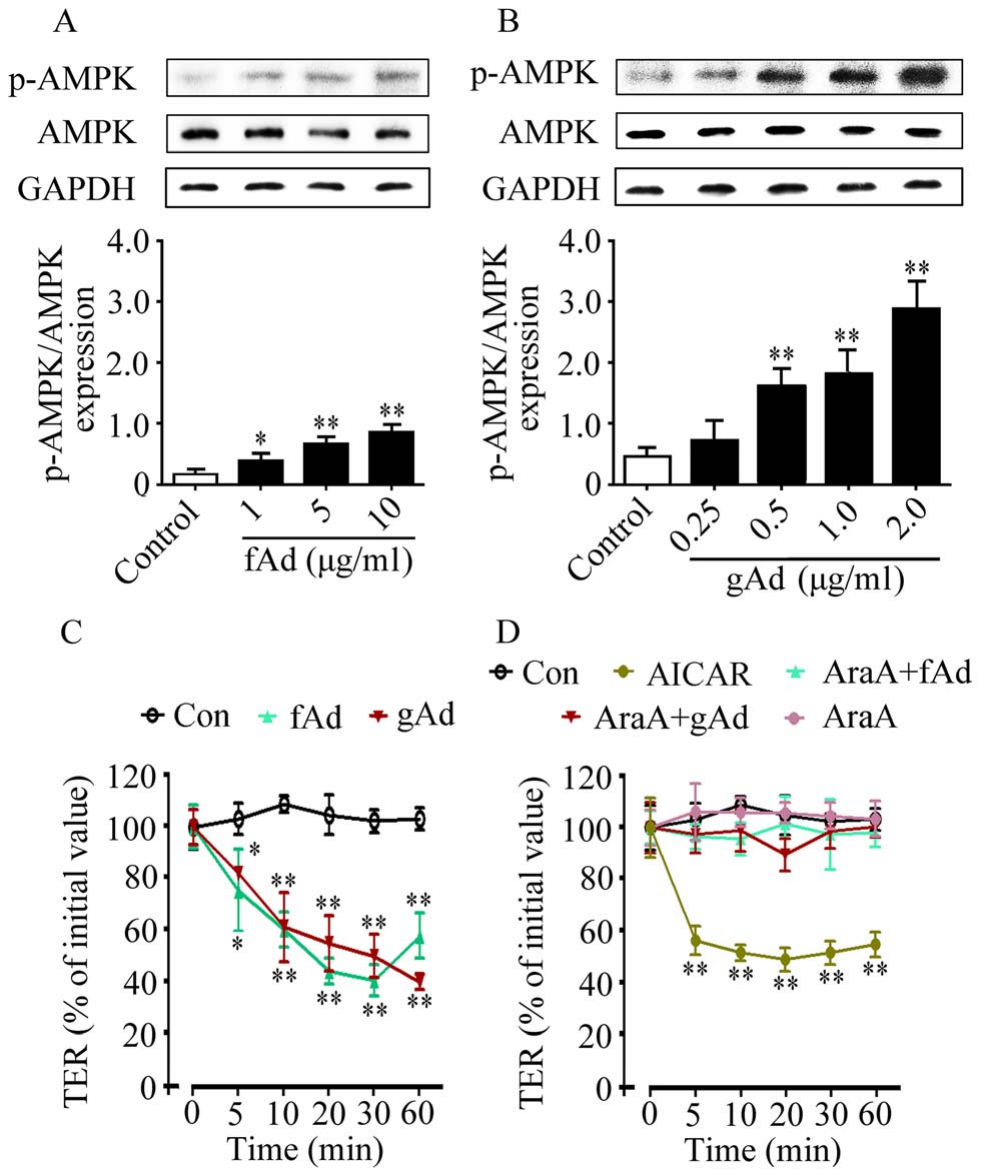
fAd and gAd decrease TER value in an AMPK-dependent manner in SMG-C6 cells. SMG-C6 cells were stimulated with 1, 5, and 10 μg/ml fAd (A) or 0.25, 0.5, 1, and 2 μg/ml gAd (B) for 10 min, respectively. The levels of p-AMPK were detected by Western blot analysis. GAPDH expression was used as an internal control. Values are means ± SD from 4 independent experiments. *P<0.05 and **P<0.01 compared with control. Time course of TER was measured by an epithelial volt ohm meter. (C) SMG-C6 cells were incubated with 5 μg/ml fAd or 1 μg/ml gAd for the indicated times. (D) SMG-C6 cells were incubated with 1 mM AICAR or treated with 1 mM AraA for 30 min and followed by fAd, gAd, or AraA alone. Values are means ± SD from 4 independent experiments. *P<0.05 and **P<0.01 compared with untreated control cells.

TER is an important indicator to evaluate the function of tight junctions, and decreased TER is associated with increased paracellular permeability [25]. To further reveal whether adiponectin-induced secretion was involved in the function of tight junctions, we next evaluated the role of adiponectin in paracellular permeability and the possible effect of AMPK. In SMG-C6 cells, fAd (1, 5, 10 μg/ml) or gAd (0.25, 0.5, 1.0, 2.0 μg/ml) treatment significantly increased the levels of p-AMPK in a dose-dependent manner (Figs. 5A and B). In untreated SMG-C6 cells, the initial TER value was 635±36.64 Ω•cm^2^, which is consistent to previous studies [26]. After incubation with 5 μg/ml fAd or 1 μg/ml gAd, TER values were significantly decreased from 5 to 60 min (Fig. 5C). 1 mM AICAR also evoked a rapid and significant drop in TER values within 60 min (Fig. 5D). Pre-treatment with AraA abolished fAd- or gAd-induced TER response, while AraA alone did not affect the TER values (Fig. 5D). These results suggested that adiponectin increased paracellular permeability in an AMPK-dependent manner.

### Both AdipoR1 and AdipoR2 are responsible for the increased AMPK phosphorylation

**Figure 6:**
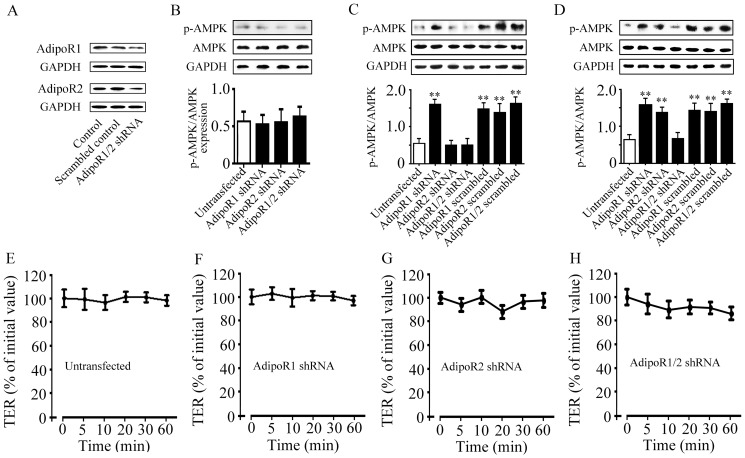
Effects of fAd and gAd on AMPK phosphorylation in SMG-C6 cells transfected with AdipoRs shRNA. (A) Expression of AdipoR1 and AdipoR2 proteins in SMG-C6 cells transfected with AdipoR1 or AdipoR2 shRNA. (B) The basal levels of p-AMPK in AdipoRs shRNA transfected cells compared with that in the untransfected cells. GAPDH expression was used as an internal control. (C) Effect of fAd on AMPK phosphorylation in SMG-C6 cells transfected with AdipoR1, AdipoR2 shRNA or scrambled controls. (D) Effect of gAd on AMPK phosphorylation in SMG-C6 cells transfected with AdipoR1, AdipoR2 shRNA or scrambled controls. Values are means ± SD from 4 independent experiments. **P<0.01 compared with untransfected cells. Time courses of TER of SMG-C6 cells without transfection (E), transfected with AdipoR1 shRNA (F), AdipoR2 shRNA (G), or AdipoRs shRNA (H). Values are means ± SD from 4 independent experiments.

To reveal the potential subtype of AdipoR that regulates AMPK phosphorylation in submandibular glands, we designed specific shRNA to knockdown the expression of AdipoR1 and AdipoR2 in SMG-C6 cells. The expression of AdipoR1 and AdipoR2 proteins was markedly reduced by 66.67% or 58.67%, respectively, compared with controls (Fig. 6A). Knockdown of AdipoR1, AdipoR2, or co-knockdown of AdipoRs did not affect the basal levels of p-AMPK (Fig. 6B) compared with that in untransfected cells. fAd stimulation for 10 min significantly increased p-AMPK level in AdipoR1 knockdown cells, but not in AdipoR2 knockdown and AdipoRs co-knockdown cells (Fig. 6C). After gAd treatment for 10 min, p-AMPK levels were significantly increased in cells transfected with either AdipoR1 or AdipoR2 shRNA, but not in AdipoRs co-knockdown cells (Fig. 6D). fAd- or gAd-induced increase in p-AMPK levels were not affected in the scrambled controls.

### Both AdipoR1 and AdipoR2 are involved in adiponectin-mediated decrease in TER

**Figure 7:**
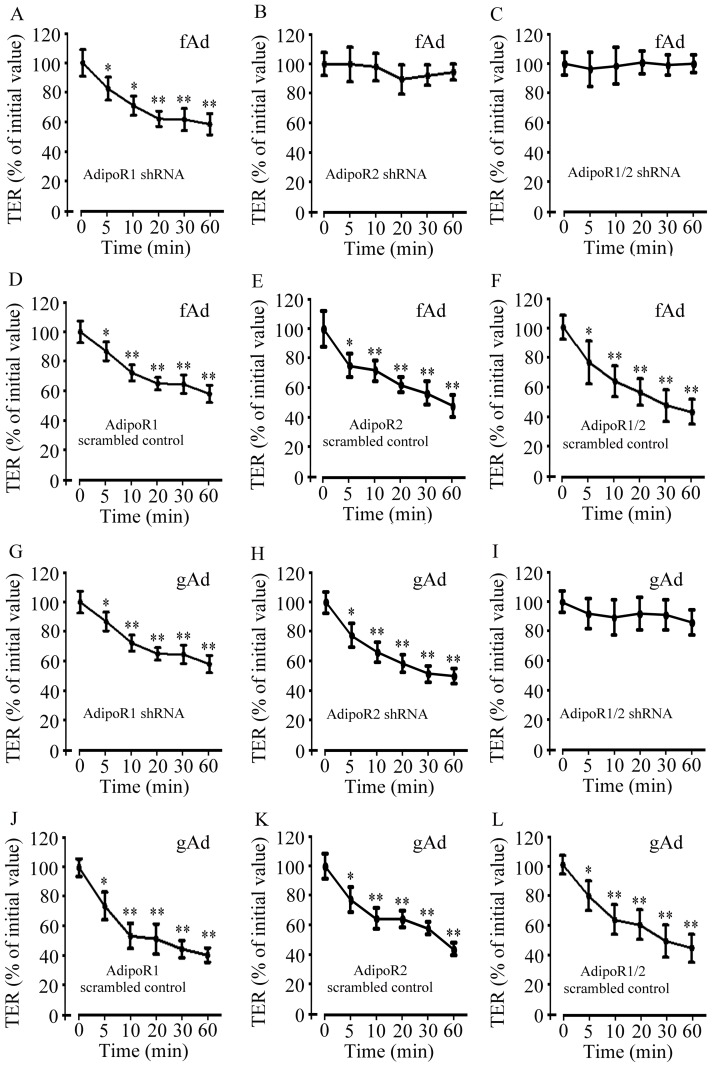
Effect of fAd and gAd on TER value in SMG-C6 cells transfected with AdipoRs shRNA. The effect of fAd on TER in SMG-C6 cells transfected with shRNA for AdipoR1 (A), AdipoR2 (B), or AdipoRs (C). The effect of fAd on TER in SMG-C6 cells transfected with scrambled controls for AdipoR1 (D), AdipoR2 (E), or AdipoRs (F), respectively. The effect of gAd on TER in SMG-C6 cells transfected with shRNA for AdipoR1 (G), AdipoR2 (H), or AdipoRs (I). The effect of gAd on TER in SMG-C6 cells transfected with scrambled controls for AdipoR1 (J), AdipoR2 (K), or AdipoRs (L). Values are means ± SD from 4 independent experiments. *P<0.05 and **P<0.01 compared with untreated cells.

The effects of AdipoR subtype on adiponectin-modulated paracellular permeability were further evaluated. Knockdown of AdipoR1, AdipoR2, or co-knockdown of AdipoRs did not affect the basal levels of TER (Figs. 6E–H). fAd significantly reduced TER values in AdipoR1 knockdown cells from 5 to 60 min (Fig. 7A), but not in AdipoR2 knockdown (Fig. 7B) or AdipoRs co-knockdown cells (Fig. 7C). fAd also significantly decreased the values of TER in the cells transfected with scrambled shRNA for AdipoR1, AdipoR2, or AdipoRs, respectively (Figs. 7D–F). In addition, gAd decreased TER values in AdipoR1 (Fig. 7G) or AdipoR2 knockdown cells (Fig. 7H), but not in AdipoRs co-knockdown cells (Fig. 7I). Transfection of scrambled shRNA for AdipoR1, AdipoR2, or AdipoRs did not alter the gAd-induced TER responses (Figs. 7J–L). Considered together, these results suggested that both AdipoR1 and AdipoR2 were responsible for the increased AMPK activation and paracellular permeability induced by adiponectin.

## Discussion

In the present study, we demonstrated that adiponectin, AdipoR1, and AdipoR2 were expressed in rat submandibular glands and characterized their distribution. Through perfusion of the isolated glands, we provided the first evidence that adiponectin functioned as a promoter of saliva secretion of submandibular glands. Furthermore, alteration of paracellular permeability was responsible for adiponectin-induced increased secretion of submandibular glands via activation of AdipoRs and AMPK. These results provided new insights into the biological role of adiponectin in salivary glands and will hopefully lead to a novel therapeutic strategy to modulate submandibular gland dysfunction.

Circulating adiponectin can regulate the metabolism and function of various cells via endocrine effect. In addition, accumulating evidence has shown that adiponectin and its receptors express ubiquitously among various organs and tissues [37]–[39], and that the locally produced adiponectin can mediate important autocrine or paracrine effects [40]. Recent research has shown that AdipoR1 and AdipoR2 mRNA exist in mouse embryos submandibular glands [17]. Expressions of adiponectin and AdipoRs also have been detected in human parotid, sublingual glands, and minor salivary glands [18], [19]. In addition, adiponectin is detected from human saliva [41], [42] and culture medium of minor salivary gland epithelial cells [19]. Here, we characterized the expression and distribution of adiponectin and AdipoRs in rat submandibular glands. These results suggested that submandibular gland epithelial cell was a new source and target of adiponectin. Adiponectin might be involved in physiological and pathophysiological processes of salivary glands via endocrine, paracrine, as well as autocrine routes.

Adiponectin is an adipokine with diverse functions. Adiponectin not only regulates energy metabolism, but also acts as an anti-inflammatory and anti-atherosclerosis factor [3]–[6]. In addition, adiponectin is involved in the regulation of apoptotic death of many types of cells [43]. Glandular epithelial cells of salivary glands are thought to play a central role in the development of saliva and autoimmune reactions. There were few studies about the role of adiponectin in oral health. A recently study has reported that adiponectin secreted from human gingival epithelial cells inhibits the up-regulation of inflammatory factors and cell injuries induced by lipopolysaccharide [44]. In addition, adiponectin suppresses of proliferation of salivary gland epithelial cells and protects cells against IFNγ-induced apoptosis [19], [20]. In the present study, we provide the first evidence that adiponectin functions as a novel promoter of saliva secretion of submandibular glands. Moreover, atropine did not inhibit the gAd-induced increase in saliva secretion, and saliva flow was further increased with carbachol combined gAd perfusion, which suggested that adiponectin promoted saliva secretion of rat submandibular glands in a non-cholinergic dependent manner.

The saliva secretion across salivary epithelium can be accomplished via either the transcellular or paracellular route [31], [45]. AQP5 plays a very important role in mediating water transport via the transcellular route in salivary glands. However, the effect of adiponectin on the expression and function of AQP5 is unknown. Here, we showed that gAd perfusion for 10 min did not alter the expression and distribution of AQP5. These results suggested that the adiponectin-induced increase in saliva secretion of rat submandibular glands was not mediated by AQP5, at least not during the early phase of induction.

Exposure to sodium caprate and pathological stimuli in Caco-2 cells results in structural alterations of tight junctions with a form of dilation, leading to enhanced paracellular permeability [35]. Another study shows that the width of apical tight junction is reduced by 50% in ClC-2^−/−^ mouse intestine, which is parallel with a significant decreased paracellular permeability [36]. In our previous studies, we found that the paracellular pathway played an important role in the secretion of rabbit submandibular glands under physiological and pathophysiological conditions [24]. In hyposecretion of rabbit transplanted submandibular glands, both the width of apical tight junctions and paracellular permeability were reduced [24]. In the present study, we found that fAd and gAd enlarged the width of apical tight junctions in submandibular gland tissues, and decreased the values of TER in SMG-C6 cells, indicating that adiponectin-induced increase in saliva secretion was mediated by modulating the structure and function of tight junction.

AMPK, a ubiquitous serine/threonine kinase, plays a key role in adiponectin-mediated metabolic modulation and cardiovascular protection [5], [10]. In MDCK cells, the activation of AMPK facilitates the assembly of tight junctions, increases TER value, and decreases the paracellular permeability [32], [33]. Activated AMPK by butyrate also enhances the intestinal barrier function by facilitating the assembly of tight junctions [34]. However, whether AMPK regulates the paracellular secretion in salivary gland epithelial cells is unknown. We found that fAd and gAd increased AMPK phosphorylation in submandibular glands. More importantly, activation of AMPK by AICAR increased saliva secretion, widened tight junction in the isolated glands, and reduced TER in cultured SMG-C6 cells. AMPK inhibitor AraA antagonized the adiponectin-induced changes of salivation, structure, and function of tight junctions. The results indicated that AMPK activation is an obligatory event in adiponectin-increased secretion and paracellular permeability in rat submandibular glands.

Adiponectin exerts its biological effects mainly through binding to AdipoR1 and AdipoR2. In skeletal muscle, both AdipoR1 and AdipoR2 contribute to the increased glucose uptake and fatty acid oxidation induced by adiponectin [10], [46]. In liver, AdipoR1 is involved in adiponectin-mediated gluconeogenesis, and AdipoR2 contributes to the increased fatty acid oxidation [10]. Although both AdipoR1 and AdipoR2 are expressed in cardiomyocytes [11], only AdipoR1 expression is decreased in rat myocardium with high-fat and high-sugar diets, possibly associated with decreased heart function [47]. The myocardial AdipoR1 level is negatively correlated with the serum insulin level [48]. AdipoR1 in the hypothalamus, brainstem, and pituitary glands relates to increased food intake, decreased energy expenditure, and the regulation of somatotrophins. In cortex, AdipoR2 is involved in controlling the cellular and behavioral action of alcohol [49]. In addition, AdiopR1-null mice have increased adiposity with augmented glucose intolerance, whereas AdipoR2-null mice are lean and resistant to diet-induced glucose intolerance [50]. These results suggest that expression and role of AdipoR1 and AdipoR2 vary with different tissues and stimuli. In salivary glands, the physiological importance of AdipoR subtype has not been explored yet. Here, we found that both AdipoR1 and AdipoR2 subtypes were expressed in submandibular glands and SMG-C6 cells. Knockdown of AdipoR1, AdipoR2, or co-knockdown of AdipoRs, respectively, did not affect the basal levels of p-AMPK and TER, which suggested that activation of AdipoRs might not be necessary for maintaining basal paracellular permeability. fAd, which binds to AdipoR1 with low affinity and AdipoR2 with intermediate affinity [11], increased p-AMKP level and decreased TER values in AdipoR1-knockdown cells, but not in AdipoR2-knockdown cells. However, gAd, which binds to AdipoR1 with high affinity, and to AdipoR2 with intermediate affinity [11], increased p-AMKP level and decreased TER values in both AdipoR1- or AdipoR2-knockdown cells. Furthermore, co-knockdown of both AdipoR1 and AdipoR2 completely antagonized the fAd- and gAd-induced AMPK and TER responses. These results suggested that both AdipoR1 and AdipoR2 were upstream molecules in adiponectin-induced signaling cascade, leading to enhanced AMPK activation and paracellular permeability in submandibular glands.

In summary, our experiments demonstrated that the rat submandibular gland was a new source and target of adiponectin. Adiponectin increased saliva secretion by modulating the structure and function of tight junctions through binding to the AdipoRs and activation of AMPK. These findings will improve our understanding of the biological role of adiponectin and provide a potential therapeutic strategy to modulate submandibular gland dysfunction.
